# Construction and Temporal Validation of a Machine Learning Model for Predicting Clinically Significant (Grade ≥2) Acute Radiation Enteritis After Radiotherapy for Cervical Cancer

**DOI:** 10.3390/jcm15218501

**Published:** 2026-11-01

**Authors:** Wenqing Hu, Xiaofeng Song, Yuanjing Wang, Jiqi Zhao, Luyao Cheng, Ming Wang, Yin Lv

**Affiliations:** 1 Department of Oncology Radiotherapy, The First Affiliated Hospital of Anhui Medical University, Hefei 230022, China; 2 Department of Radiation Therapy Center, Beijing Obstetrics and Gynecology Hospital, Capital Medical University, Beijing Maternal and Child Health Care Hospital, Beijing 100006, China; 3 Department of Gynecological Oncology, Beijing Obstetrics and Gynecology Hospital, Capital Medical University, Beijing Maternal and Child Health Care Hospital, Beijing 100006, China

**Keywords:** cervical cancer, concurrent chemoradiotherapy, acute radiation enteritis, machine learning, SHAP

## Abstract

**Purpose:** To develop and temporally validate prediction models for clinically significant acute radiation enteritis, defined as Common Terminology Criteria for Adverse Events (CTCAE) grade ≥2 ARE and hereafter abbreviated SARE to remain consistent with the published literature on this endpoint, in patients with cervical cancer undergoing pelvic radiotherapy. **Methods:** A total of 363 patients diagnosed with cervical cancer were retrospectively included and received concurrent chemoradiation at the First Affiliated Hospital of Anhui Medical University. The training set (*n* = 203, 70%) and the internal test set (*n* = 87, 30%) were recruited from January 2020 to January 2024; an independent temporal validation cohort (*n* = 73; 72 with complete outcome and prediction data) was recruited from January 2024 to December 2025. Feature selection (Boruta plus random-forest recursive feature elimination) and hyperparameter tuning were performed exclusively within the training set. Six models were constructed—logistic regression (LR), a multilayer perceptron (MLP), a support vector machine (SVM), random forest (RF), XGBoost and LightGBM—and SHapley Additive exPlanations (SHAP) was used to interpret the final model. **Results:** Twelve optimal predictors were selected. The LR model demonstrated the most balanced performance, achieving AUCs of 0.855 (95% CI 0.792–0.910) in the training set, 0.830 (95% CI 0.721–0.918) in the internal test set, and 0.753 (95% CI 0.621–0.864) in the temporal external validation cohort. RF and LightGBM fitted the training data perfectly (AUC 1.000) but fell to 0.762 and 0.757 in validation, and XGBoost, the SVM and the MLP were lower (0.661, 0.599 and 0.616). In temporal validation LR had the lowest Brier score (0.176) but a sensitivity of only 0.40 (95% CI 0.176–0.619) at the 0.5 threshold. Decision curve analysis showed positive net benefit for LR across threshold probabilities of 0.05–0.80. SHAP ranked Rectal_Dmean, Bowel_V30 and SII as the leading contributors. **Conclusions:** Dosimetric parameters combined with composite inflammatory–nutritional indices show promising discrimination for grade ≥2 ARE. The LR model provides a simple, interpretable tool for pre-radiotherapy risk stratification, but prospective, multicenter validation is required before routine clinical implementation.

## 1. Introduction

Cervical cancer remains the fourth most common malignancy among women worldwide [1,2]. For patients with locally advanced or postoperative high-risk disease, radiotherapy (RT) is a cornerstone treatment that improves local control and survival. However, the radiosensitivity of normal intestinal tissues within the pelvis often leads to acute radiation enteritis (ARE), which is one of the most frequent and dose-limiting toxicities [3,4,5].

ARE typically occurs during RT or within three months after completion, manifesting as diarrhea, abdominal pain, tenesmus, and bloody stools. We use the term clinically significant ARE for Common Terminology Criteria for Adverse Events (CTCAE) v5.0 grade ≥2 toxicity, which typically requires clinical interventions such as antidiarrheals, fluid resuscitation, nutritional support, or even treatment interruption, and may thereby compromise tumor control [6,7,8]. Early identification of patients at high risk for grade ≥2 ARE is therefore clinically important.

Multiple factors have been associated with severe ARE, including patient characteristics (age, comorbidities, nutritional status), tumor-related factors (stage, diameter), treatment variables (RT dose, concurrent chemotherapy), dosimetric parameters (especially rectal and small bowel V20–V40), and inflammatory–immune–nutritional indices (NLR, PLR, SII, PNI, etc.) [7,8,9,10,11,12]. Most existing predictive models rely on traditional logistic regression or nomograms, which may fail to capture complex non-linear relationships and interactions among these variables [7,8,9].

Machine learning (ML) offers advantages in handling high-dimensional, multi-source data and can model non-linear associations while mitigating overfitting through cross-validation and regularization. Several ML algorithms—such as random forest (RF), XGBoost, support vector machines (SVMs), and gradient boosting machines—have been applied to radiation toxicity prediction [13,14,15]. However, studies specifically targeting severe ARE and systematically integrating dosimetric, inflammatory, and nutritional parameters remain limited.

In this study, we constructed and compared six ML models (logistic regression, random forest, an SVM, XGBoost, LightGBM, and a multilayer perceptron) to predict grade ≥2 ARE in cervical cancer patients undergoing radiotherapy. We aimed to select the model with the best discriminative ability and generalizability, and to interpret the optimal model using SHAP analysis, thereby providing a tool for pre-treatment risk stratification, RT plan optimization, and individualized supportive care. All model and feature selection was performed within the development cohort, and the independent cohort recruited in a later calendar period was reserved for a single temporal validation of the final pre-specified model.

## 2. Materials and Methods

### 2.1. Study Population and Data Collection

This study retrospectively included 363 patients who were diagnosed with cervical cancer and received concurrent chemoradiation in the First Affiliated Hospital of Anhui Medical University from January 2020 to December 2025. This study was approved by the Institutional Ethics Committee (PJ-23 April 2024), and the requirement for informed consent was waived due to the retrospective nature of the analysis.

Inclusion criteria were: (1) histopathologically confirmed cervical cancer; (2) age ≥18 years and ≤80 years; (3) stage IB1–IVA according to the 2018 FIGO staging system; (4) complete clinical, laboratory, and RT planning dosimetric data available before treatment; (5) acute gastrointestinal toxicity assessment records available during RT and for at least three months after RT completion; (6) first course of pelvic RT. Exclusion criteria were: (1) prior pelvic or abdominal RT; (2) pre-existing inflammatory bowel disease, intestinal obstruction, fistula, perforation, severe chronic diarrhea, or other conditions affecting ARE assessment before RT; (3) coexisting active malignancy; (4) severe infection, severe hepatic/renal insufficiency, hematologic disease, or immune system disease that could significantly affect peripheral blood inflammatory markers before treatment; (5) incomplete prescribed RT, or RT interruption >7 consecutive days for reasons other than ARE (the reason for every interruption is reported in Section 3.1); (6) excessive missing data for key variables, precluding reliable imputation or modeling.

Of the whole cohort, the 290 patients recruited from January 2020 to January 2024 constituted the development cohort and were randomly split into a training set (70%, *n* = 203) and an internal test set (30%, *n* = 87) using stratified sampling to preserve the SARE proportion. An independent temporal validation cohort of 73 patients was recruited from January 2024 to December 2025; 72 of these 73 patients had complete outcome and prediction data and were included in the performance analysis. Throughout this manuscript this cohort is termed the temporal validation cohort rather than an external validation cohort, because it was recruited at the same institution and therefore provides temporal, but not geographic or inter-institutional, validation.

### 2.2. Treatment Protocol

#### 2.2.1. Radiotherapy

All patients received intensity-modulated radiotherapy (IMRT). Before CT simulation, patients were instructed to empty the bladder and rectum and drink 500 mL of warm water to fill the bladder. Patients were positioned supine and immobilized with a thermoplastic mask. CT simulation was performed from the lower edge of T10 to 5 cm below the ischial tuberosity with 5 mm slice thickness. CT images were transferred to the Eclipse (version 13.6) treatment planning system for target delineation and planning. The gross tumor volume (GTV) included the primary tumor, involved adjacent structures, and metastatic lymph nodes as shown on gynecologic examination and imaging. The clinical target volume (CTV) included the GTV, cervix, uterus, parametrial tissues, adnexa, posterior bladder wall, involved vaginal areas, and pelvic lymphatic drainage areas. Para-aortic or inguinal lymphatic drainage areas were included when necessary. The planning target volume (PTV) was formed by expanding the CTV by 0.5–2.0 cm to account for setup errors and organ motion. Organs at risk (OARs) included the bladder, rectum, small bowel, colon, spinal cord, pelvic bones, and bilateral femoral heads.

The prescribed dose to the pelvic PTV was 50.0–50.4 Gy in 25–28 fractions. Positive lymph nodes received a boost to the involved-node PTV (PTV-nd) of 60 Gy in 30 fractions. Treatment plans were evaluated using dose–volume histograms (DVHs) for target coverage and OAR sparing, meeting the following constraints: bladder V50 ≤ 50%, rectum V50 ≤ 50%, small bowel V50 ≤ 10%, bilateral femoral heads V50 ≤ 5%, and spinal cord V40 = 0%. External-beam RT was delivered using 6 MV X-rays with a 7-field IMRT technique.

When the external-beam cumulative dose reached approximately 45 Gy, weekly intracavitary brachytherapy (ICBT) was interspersed. ICBT used a three-dimensional afterloading technique with a dose reference point set 0.5 cm below the vaginal mucosa, targeting the cervix and all visible residual tumor. A total of 4–5 ICBT fractions of 5–6 Gy each were delivered.

#### 2.2.2. Concurrent Chemotherapy

Based on patient pathology stage, age, and general condition, concurrent chemotherapy consisted of either platinum monotherapy or platinum combined with paclitaxel. Doses were determined and adjusted according to National Comprehensive Cancer Network (NCCN) guidelines.

### 2.3. Data Collection and Follow-Up

The clinical and treatment information collected during the study included demographic and clinical data such as age, body mass index (BMI), hypertension, diabetes, clinical stage, surgery history, concurrent chemotherapy cycles, tumor diameter, histologic type, RT planning, dosimetric parameters of RT, and RT duration.

Dose–volume histogram parameters of radiotherapy were exported from the treatment planning system. Organs at risk included the rectum and small bowel. Rectal contours followed departmental guidelines, typically from the superior anal canal/sciatic tuberosity level to the rectosigmoid junction. The small bowel was contoured using the bowel loop method (contouring individual bowel loops) according to departmental practice, with contouring consistency verified by two experienced radiation oncologists. All plans were reviewed by experienced radiation oncologists or physicists to ensure dosimetric consistency [3,6].

Dose–volume parameters, defined as the percentage of organ volume receiving a specified dose, were recorded as Vx. Recorded parameters included Rectal_V20, Rectal_V30, Rectal_V40, Rectal_V45, Rectal_V50, Bowel_V20, Bowel_V30, Bowel_V40, Bowel_V45, Bowel_V50, Rectal_Dmean, and Bowel_Dmean. All rectal and bowel dose–volume parameters described above were exported from the treatment planning system for the external-beam plan. The intracavitary brachytherapy contribution was not incorporated into the dosimetric predictors used for modeling; because more than 80% of this retrospective cohort was treated with conventional two-dimensional intracavitary brachytherapy prescribed to reference points (point A and the ICRU rectal and bladder points), three-dimensional rectal and bowel DVH parameters for the brachytherapy component could not be reconstructed reliably for the whole cohort.

### 2.4. Laboratory Biomarkers

Peripheral venous blood samples were collected within one week before treatment (prior to both RT and chemotherapy initiation). Hematologic parameters included neutrophil count (NE, ×10^9^/L), monocyte count (MONO, ×10^9^/L), lymphocyte count (LY, ×10^9^/L), and hemoglobin (HB, g/L). Biochemical parameters included lactate dehydrogenase (LDH, U/L) and albumin (ALB, g/L). Derived inflammatory–immunonutritional indices were calculated as follows:

NLR = neutrophil count/lymphocyte count;
PLR = platelet count/lymphocyte count;
LMR = lymphocyte count/monocyte count;
SIRI = neutrophil count × monocyte count/lymphocyte count;
SII = platelet count × neutrophil count/lymphocyte count;
PNI = albumin (g/L) + 5 × lymphocyte count (10^9^/L);
LAR = lactate dehydrogenase/albumin.

### 2.5. Outcome Definition and the Grading of Acute Radiation Enteritis

The primary outcome was development of grade ≥2 acute radiation enteritis during RT or within three months after RT completion. ARE was graded according to the Common Terminology Criteria for Adverse Events (CTCAE) version 5.0 [16]. Diarrhea was graded as follows: grade 1, increase of <4 stools per day over baseline; grade 2, increase of 4–6 stools per day over baseline and/or limiting instrumental activities of daily living (ADL); grade 3, increase of ≥7 stools per day over baseline, hospitalization indicated, and/or limiting self-care ADL; grade 4, life-threatening consequences with urgent intervention indicated; and grade 5, death. Consistent with previous studies [8,12] and because grade ≥2 toxicity typically requires clinical intervention, grade 0–1 was defined as non-clinically significant ARE (non-SARE) and grade ≥2 as severe/clinically significant ARE (SARE). All outcome grades were re-verified against the source medical records during this revision. To minimize inter-observer variability, all grades were assigned by the same two radiation oncologists, with discrepancies resolved by consensus.

### 2.6. AI Use Disclosure

Kimi-k2.7 was used for checking for spelling and grammar errors, and running a plagiarism check.

### 2.7. Statistical Analysis

Continuous variables were compared using an independent t-test (normal distribution) or the Mann–Whitney U test (non-normal). Normality was assessed using the Shapiro–Wilk test. Categorical variables were compared using χ^2^ or Fisher’s exact test.

Prior to modeling, continuous predictors were standardized (z-score normalization); scaling parameters were estimated exclusively within the training set and then applied unchanged to the internal test and validation cohorts. Missing values were handled by complete-case analysis, as overall missingness was low (<2% per predictor; no predictor exceeded 2% missing values). The random seed was fixed (random_state = 42) for data splitting, hyperparameter search, and model fitting to ensure full reproducibility. Class imbalance (SARE prevalence 29.6%) was addressed by stratified sampling and by evaluating precision–recall curves in addition to ROC curves; no synthetic oversampling was applied.

Feature selection was performed using the Boruta algorithm followed by random-forest-based recursive feature elimination (RFE) [17,18]. Both steps were conducted exclusively within the training set using 5-fold cross-validation to prevent data leakage. The stopping criterion for RFE was a decline in the cross-validated AUC of >0.01. Six models were developed: logistic regression (LR), random forest (RF) [19], a support vector machine (SVM) [20], XGBoost [21], LightGBM [22], and a multilayer perceptron (MLP). The optimal model was interpreted using SHAP values [23]. This study followed the TRIPOD and TRIPOD+AI reporting guidelines [24,25]. Model selection was performed exclusively using the development data (internal test performance, calibration, and interpretability); the temporal validation cohort was never used for feature selection, hyperparameter tuning, or model selection, and was evaluated only once for the final pre-specified model. The final pre-specified LR model—the same 12 predictors and the same hyperparameters selected within the training set—was subsequently refitted using all 290 patients of the development cohort, and the temporal validation cohort was then evaluated once with this refitted model.

Hyperparameter tuning was performed using grid search with 5-fold cross-validation within the training set, optimizing the mean cross-validated AUC. The search ranges were: LR, C ∈ {0.03, 0.1, 1, 10}, L2 penalty, solvers lbfgs/saga; RF, 500 trees, max_depth = 1, min_samples_split = 2, min_samples_leaf = 5, max_features = 3, max_samples = 0.3, ccp_alpha ∈ {0, 0.005, 0.03}; SVM, C ∈ {0.1, 1, 10}, RBF kernel, gamma ∈ {scale, auto}; XGBoost, learning_rate = 0.01, max_depth = 1, n_estimators = 10, min_child_weight ∈ {1, 3, 5}, reg_lambda = 1, max_bin = 4, subsample = 1, colsample_bytree = 0.5; LightGBM, learning_rate = 0.01, num_leaves = 2, max_depth = 1, n_estimators = 20, min_child_samples = 20, reg_alpha = 1, reg_lambda ∈ {10, 50}, feature_fraction = 0.8, bagging_fraction = 0.5, bagging_freq = 1, boosting_type = gbdt; MLP, hidden_layer_sizes ∈ {(50,), (100,), (100, 50)}, learning_rate_init ∈ {0.001, 0.01}, max_iter ∈ {200, 300}. The 70:30 stratified split and model construction used random_state = 42; RFE used 5-fold splits with random_state = 42; Boruta was run 20 times with seeds 0–19. The recomputation environment was Python 3.11.9, NumPy 1.26.4, pandas 2.3.3, scikit-learn 1.7.2, XGBoost 2.0.3, LightGBM 4.6.0, and SHAP 0.45.1; the original BorutaPy version was not recorded. Class proportions were preserved by stratified splitting; the final six classifiers did not use oversampling, undersampling, or class weights—only the random forest used during the Boruta screening stage employed balanced class weights. Confusion-matrix metrics were calculated using a classification threshold of 0.5.

Model performance was assessed using the area under the receiver operating characteristic curve (AUC; 95% CIs by DeLong’s method and 2000 stratified bootstrap resamples), precision–recall curves, calibration curves (Brier score, calibration intercept, and calibration slope), and decision curve analysis. Accuracy, sensitivity, specificity, precision, and F1 score were calculated at a classification threshold of 0.5; DCA was evaluated over a threshold probability range of 0.05–0.80, encompassing the risk levels at which intensified monitoring or prophylactic supportive care would be considered clinically reasonable. A two-sided *p* < 0.05 was considered statistically significant. Analyses were performed using SPSS 27.0, R 4.4.0, and Python 3.12.5. A fixed probability threshold of 0.5 was used for the confusion-matrix metrics because it is the conventional default and yields a single pre-specified, interpretable operating point; no outcome-weighted or clinically derived threshold was available before validation. Because a threshold of 0.5 may be inappropriate when the intended use is to identify patients at increased risk, the trade-off between sensitivity and specificity across thresholds is also reported, and the choice of threshold is discussed as a limitation. The DCA threshold range of 0.05–0.80 was pre-specified to encompass the risk levels at which intensified gastrointestinal monitoring or prophylactic supportive care would be considered clinically reasonable at our institution; below 0.05 the intervention would be offered to almost everyone and above 0.80 to almost no one, so net benefit outside this range is not interpretable in practice. Net benefit on DCA is reported as evidence about the consequence of using the model at a given threshold, and is interpreted separately from discrimination and calibration rather than as a summary measure of clinical utility.

## 3. Results

### 3.1. Patient Characteristics and Feature Selection

**Figure 1:**
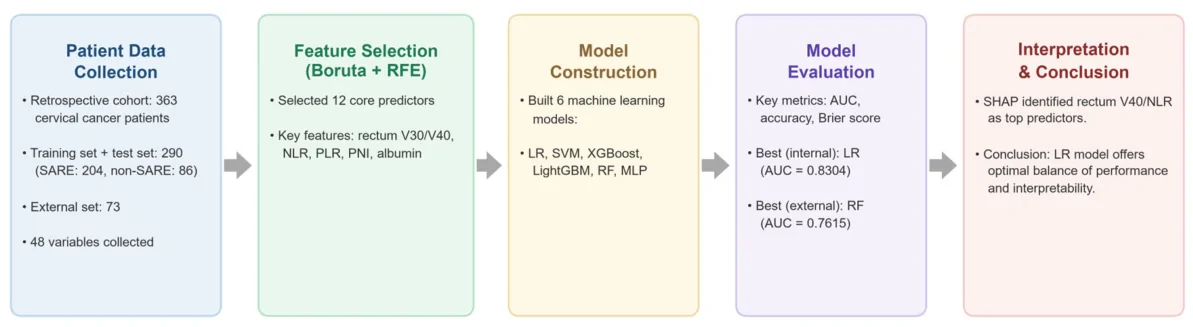
Study flow and workflow for development and temporal validation of the grade ≥2 acute radiation enteritis risk prediction model.

Of 371 patients screened, 363 were included and eight were excluded because radiotherapy was interrupted for more than 7 consecutive days. The reason for each of these eight interruptions was re-reviewed against the source medical records: six were due to severe hematologic toxicity, one to a severe non-gastrointestinal infection, and one to treatment discontinuation for financial reasons. None of the eight interruptions was caused by gastrointestinal toxicity, and no patient was excluded because of ARE. The 290 included patients were randomly split into a training set (70%, *n* = 203; SARE events, *n* = 60) and an internal test set (30%, *n* = 87; SARE events, *n* = 26), together with an independent temporal validation cohort (*n* = 73; SARE events, *n* = 20). The incidence of SARE was 29.6% (86/290) in the development cohort, with 60 events in the training set and 26 in the internal test set, while the remaining 70.4% (204/290) had grade 0–1 ARE. With 86 events and 12 final predictors, the crude events-per-variable (EPV) ratio for the model refitted on the entire development cohort was 7.2; if the 60 training-set events alone are used, the crude ratio is 5.0. A total of 48 candidate predictors were screened and the final set was chosen by a data-driven procedure, so the effective number of degrees of freedom consumed exceeds 12. The moderate bootstrap selection stability reported below (mean Jaccard 0.560) and the calibration slope of 0.598 observed in temporal validation both indicate that the model remains at some risk of overfitting. Seventy-two of the 73 patients in the temporal validation cohort had complete outcome and prediction data and were included in the performance analysis, with 20 SARE events (baseline characteristics are shown in Table S1). The overall workflow is illustrated in Figure 1.

Baseline clinical, dosimetric, and laboratory characteristics of the SARE and non-SARE groups are summarized in Table 1. Compared with the non-SARE group, patients with SARE had significantly higher rates of hypertension, larger tumor diameter, poorer tumor differentiation, and deeper infiltration, and were less likely to have undergone prior surgery. Dosimetrically, the SARE group exhibited significantly higher rectal and small bowel doses across multiple parameters, including Rectal_Dmean, Rectal_V30, Rectal_V40, Bowel_Dmean, and Bowel_V30 (all *p* < 0.05). Inflammatory and nutritional indices also differed markedly: the SARE group had elevated NLR, PLR, SII, and LAR, and reduced albumin and PNI (all *p* < 0.01). No significant differences were observed in age, BMI, diabetes, or most chemotherapy-related variables.

**Figure 2:**
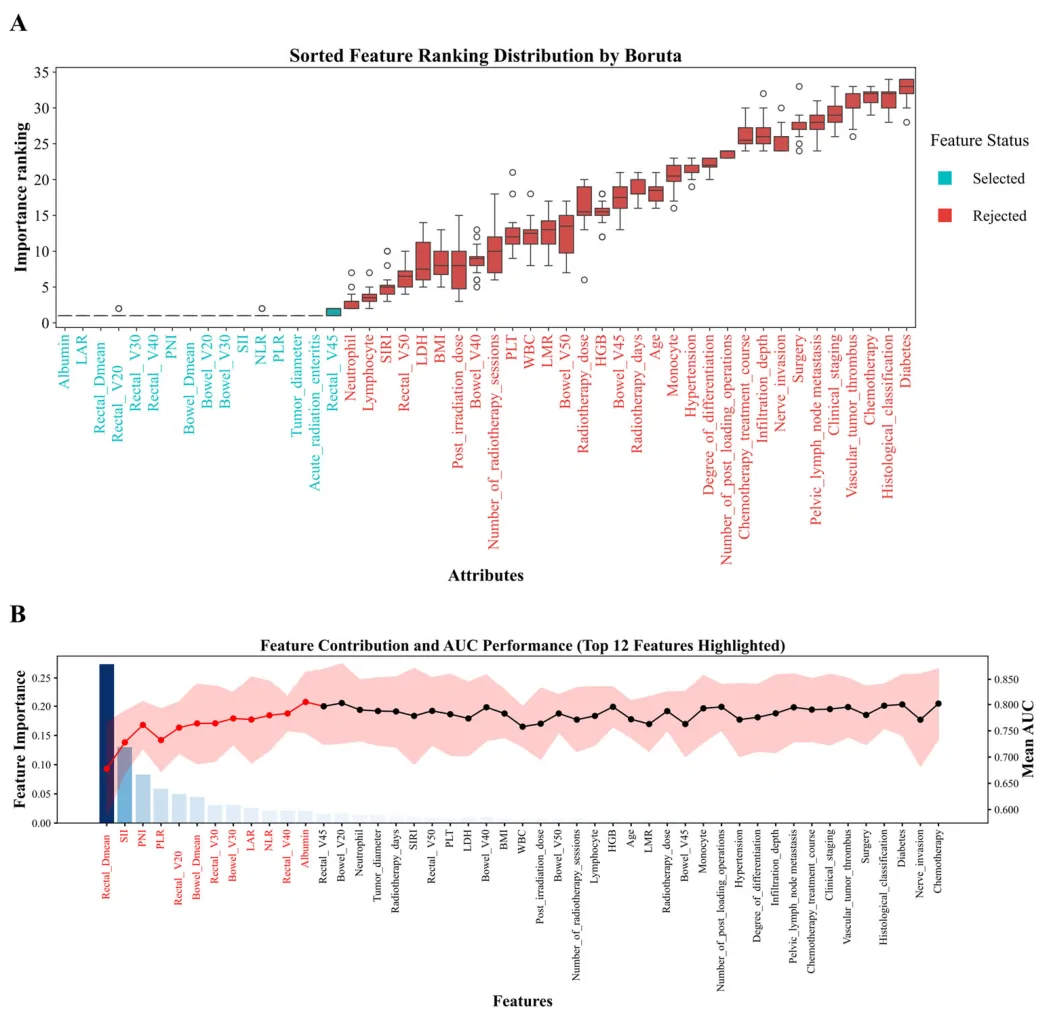
Feature selection for predicting severe acute radiation enteritis. (**A**) Boruta algorithm output showing variable importance ranking. Green boxplots denote confirmed important variables (e.g., albumin, LAR, Rectal_Dmean, Rectal_V20, Rectal_V30, Rectal_V40, PNI, Bowel_Dmean, Bowel_V20, Bowel_V30, SII, NLR, PLR). Red boxplots represent rejected variables, respectively. The dashed horizontal line indicates the maximum importance of shadow features. (**B**) Recursive feature elimination (RFE) results based on random forest. Bar heights represent the contribution weight of each variable, and the overlaid line plot shows the cumulative AUC as variables are added. The red bars indicate the final 12 selected predictors: Rectal_Dmean, SII, PNI, PLR, Rectal_V20, Bowel_Dmean, Rectal_V30, Bowel_V30, LAR, NLR, Rectal_V40, and albumin.

To identify the most relevant predictors, feature selection was performed using the Boruta algorithm combined with random-forest-based recursive feature elimination (RFE). The Boruta algorithm identified 13 variables as confirmed important, including albumin, LAR, Rectal_Dmean, Rectal_V20, Rectal_V30, Rectal_V40, PNI, Bowel_Dmean, Bowel_V20, Bowel_V30, SII, NLR, and PLR (Figure 2A). Subsequent RFE based on variable contribution and cumulative AUC changes selected a final set of 12 predictors for model development: Rectal_Dmean, SII, PNI, PLR, Rectal_V20, Bowel_Dmean, Rectal_V30, Bowel_V30, LAR, NLR, Rectal_V40, and albumin (Figure 2B). The stability of the selected predictor set was assessed by bootstrap resampling (1000 resamples), showing a mean Jaccard similarity of 0.560 (standard deviation 0.108, median 0.600, range 0.333–1.000). Multicollinearity among the final predictors was assessed using the variance inflation factor (VIF); all VIFs were below the conventional threshold of 10 (maximum VIF: 9.11, for PNI), supporting the stability of the LR coefficients (Table S2). A mean Jaccard similarity of 0.560 (range 0.333–1.000) indicates only moderate stability of the selected predictor set rather than the stable, reproducible panel that a higher value would imply, and the maximum VIF of 9.11 (for PNI) indicates substantial correlation among several predictors, particularly the multiple correlated rectal dose metrics and the overlapping inflammatory and nutritional indices. These findings do not invalidate the penalized LR model as a prediction tool, but they make the individual coefficients and the SHAP ranking interpretable only with caution; bootstrap selection frequencies and the uncertainty around the coefficients are reported in Table S2 and should be inspected before any single predictor is interpreted in isolation.

### 3.2. Model Performance Comparison

**Figure 3:**
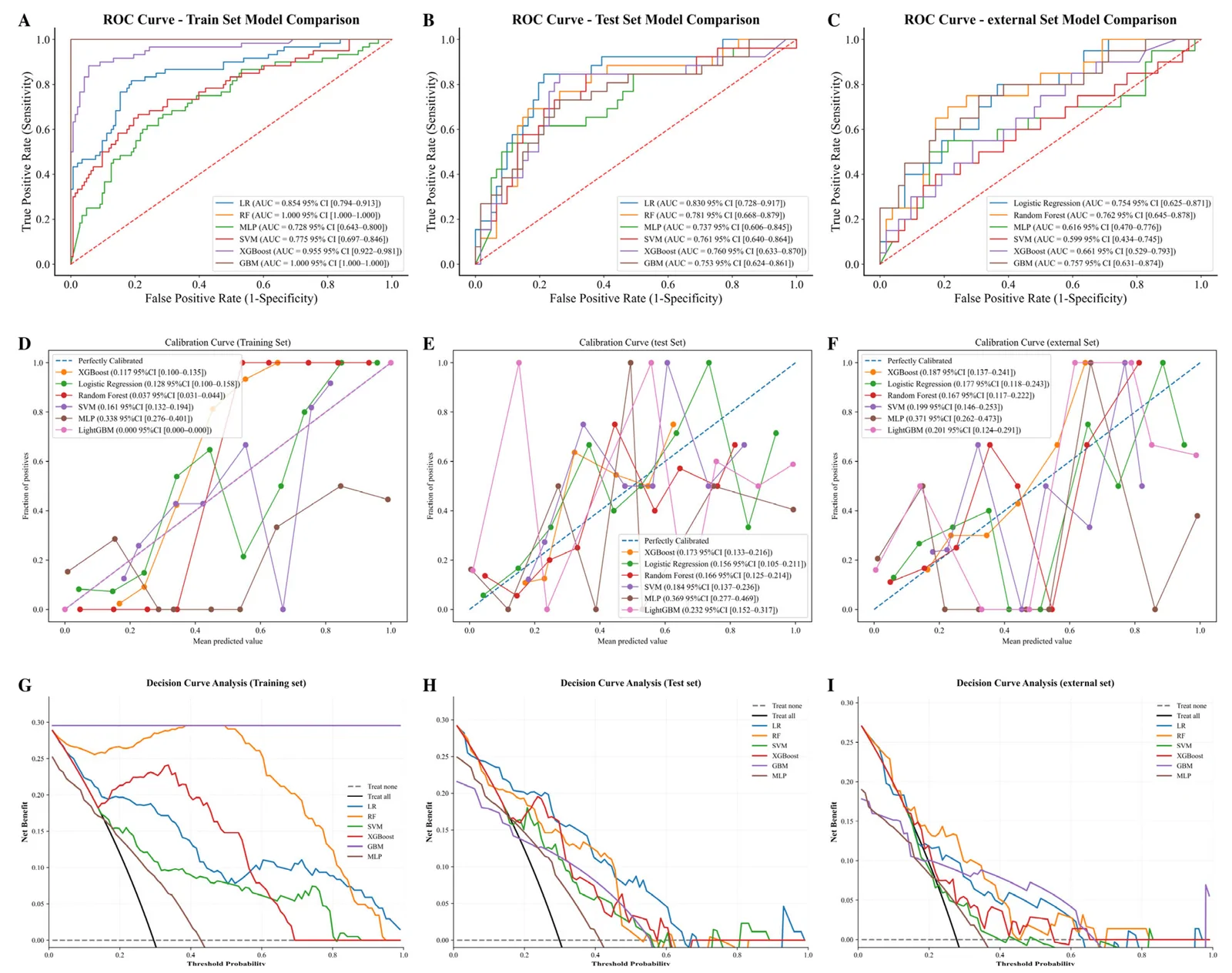
Discriminative performance of six machine learning models across training, internal test and temporal validation sets. Rows represent the three datasets: training set (**A**,**D**,**G**), internal test set (**B**,**E**,**H**), and temporal validation cohort (**C**,**F**,**I**). Columns display different performance metrics: receiver operating characteristic (ROC) curves with AUC values (**A**–**C**), calibration curves with Brier scores (**D**–**F**), and decision curve analysis (DCA) showing net benefit across threshold probabilities (**G**–**I**). The logistic regression (LR) model demonstrates the most stable discrimination and calibration, while ensemble models (RF, LightGBM) show overfitting with a perfect training AUC but lower temporal validation performance.

Six machine learning models—logistic regression (LR), random forest (RF), a support vector machine (SVM), XGBoost, LightGBM, and a multilayer perceptron (MLP)—were trained and validated. Their discriminative performance was evaluated using the area under the receiver operating characteristic curve (AUC) and precision–recall curves, along with calibration curves (Brier score, calibration intercept, and calibration slope) and DCA across the training, internal test, and temporal validation sets (Figure 3; Table 2). The LR model demonstrated the most stable and balanced performance across all datasets. It achieved an AUC of 0.855 (95% CI: 0.792–0.910) in the training set, 0.830 (95% CI: 0.721–0.918) in the internal test set, and 0.753 (95% CI: 0.621–0.864) in the temporal validation cohort. At the 0.5 classification threshold, the corresponding sensitivity and specificity were 0.500 and 0.902 in the training set, 0.577 and 0.869 in the internal test set, and 0.400 (95% CI 0.176–0.619) and 0.904 in the temporal validation cohort, identical to the values reported in Table 2. Calibration was acceptable in the development data but not in the temporal validation cohort. Brier scores were 0.128, 0.153 and 0.176, calibration intercepts 0.055, −0.187 and −0.200, and calibration slopes 1.076, 0.697 and 0.598 in the training, internal test and temporal validation cohorts, respectively. A calibration slope of 0.598, substantially below 1, indicates that the temporal validation predictions were too extreme and is evidence of residual overfitting rather than satisfactory calibration; we therefore do not describe calibration in the temporal validation cohort as satisfactory. Decision curve analysis showed positive net benefit for the LR model over the pre-specified threshold probability range (0.05–0.80) (Figure 3G–I; Table S3), which we interpret as evidence about the consequences of using the model at those thresholds, and not, on its own, as evidence of discrimination, of calibration, or of clinical utility.

In contrast, ensemble models such as RF and LightGBM showed perfect discrimination in the training set (AUC = 1.000) but exhibited substantial performance drops in validation: RF achieved internal test and temporal validation AUCs of 0.781 and 0.762, respectively, while LightGBM achieved 0.753 and 0.757, indicating clear overfitting. The perfect training-set discrimination of RF and LightGBM is characteristic of high-variance ensemble methods, which can fully separate training samples in-sample; because hyperparameters were selected by cross-validation rather than by training-set fit, the chosen configurations memorize training-set noise and generalize poorly, as reflected by the marked decline in the independent cohorts. XGBoost showed moderate performance (internal test AUC = 0.760, temporal validation AUC = 0.661) but with poor calibration (Brier scores up to 0.241). The SVM and MLP yielded lower overall performance, with temporal validation AUCs of 0.599 and 0.616, respectively, and suboptimal calibration (Table S4).

In terms of accuracy, sensitivity and F1 score, the LR model again ranked among the best, achieving an internal test set accuracy of 78.2%, sensitivity of 57.7% and F1 score of 0.612 (Table 2). In the temporal validation cohort the same model achieved an accuracy of 76.4% and an F1 score of 0.485, with a sensitivity of 0.400 (95% CI 0.176–0.619) and a specificity of 0.904; these values are identical to those reported in Table 2, which was regenerated from a single locked prediction vector and is now the sole source of all performance figures quoted in the text. These findings indicate that complex tree-based models may achieve high in-sample fit but generalize poorly at this sample size, whereas the more parsimonious LR model was more robust across cohorts. Precision–recall curves supported the same model ranking (Figure S1).

### 3.3. Feature Importance and Model Interpretation Using SHAP

**Figure 4:**
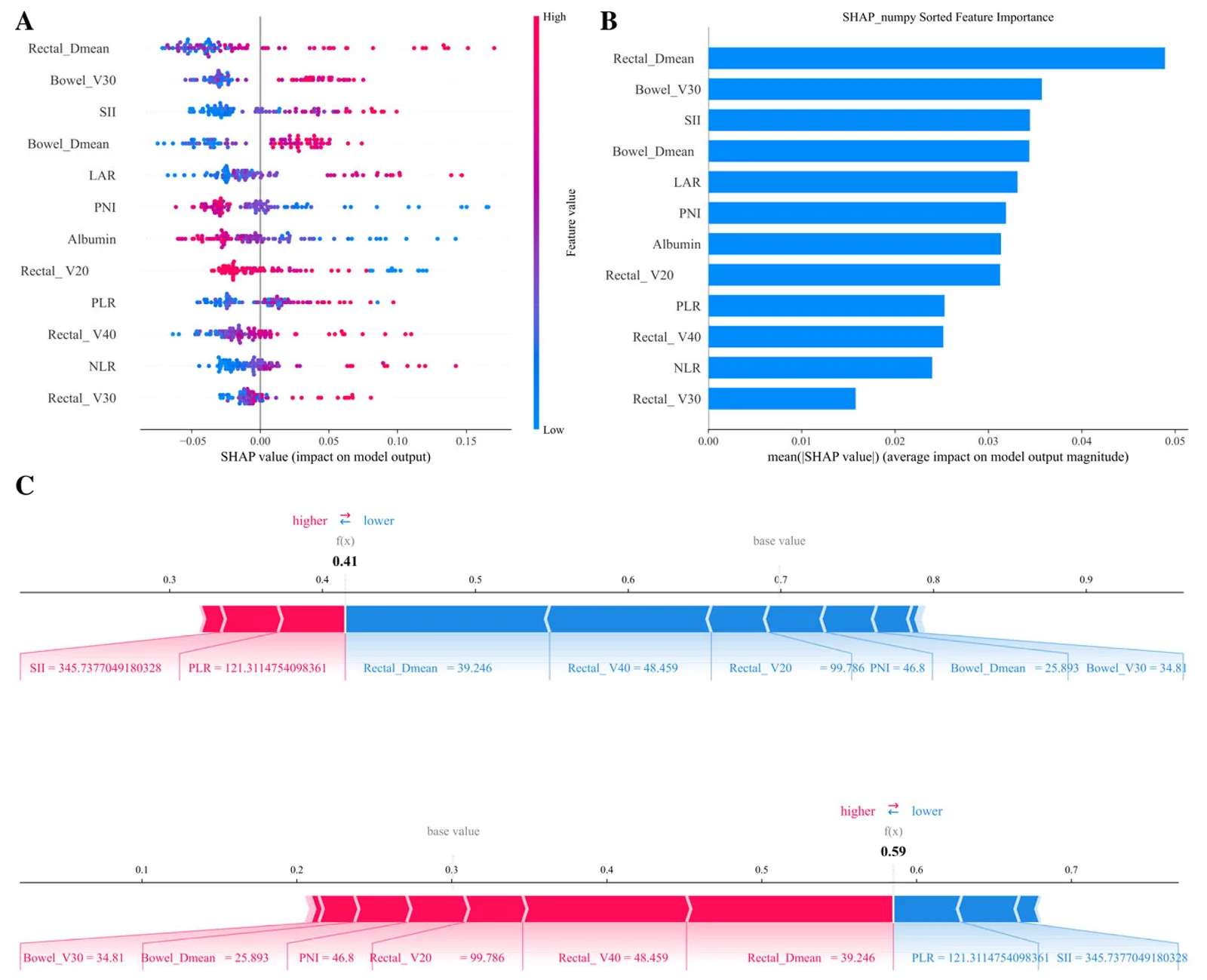
SHAP-based interpretability of the logistic regression model for grade ≥2 ARE prediction. (**A**) SHAP summary (beeswarm) plot: Each point represents a SHAP value for a given feature across all patients. The *x*-axis indicates the impact on model output (positive SHAP = higher risk), and the color gradient (red to blue) reflects the feature value from high to low. Features are ordered by mean absolute SHAP value, with Rectal_Dmean, Bowel_V30 and SII being the top three contributors. Because the final model is a linear logistic regression, each SHAP value is a linear function of the corresponding feature value (SHAPi = βi·(xi − E[xi])); the plot therefore reflects the direction and relative magnitude of each association and implies no non-linear or threshold effect for any predictor. (**B**) Bar plot of mean absolute SHAP values of the 12 predictors. (**C**) Force plots for two representative patients from the internal test set: the left panel shows a low-risk patient (predicted probability 0.41) with protective feature contributions; the right panel shows a high-risk patient (predicted probability 0.59) where elevated dosimetric and inflammatory indices drive the prediction toward grade ≥2 ARE.

To enhance interpretability, we applied SHapley Additive exPlanations (SHAP) to the final LR model. The SHAP summary plot (Figure 4A) shows the contribution of each feature to the model’s output, ranked by mean absolute SHAP value. Consistent with Figure 4A, Rectal_Dmean, Bowel_V30, and SII were the top three contributors; Rectal_V30 showed the smallest mean absolute SHAP contribution. Higher values of dosimetric and inflammatory indices generally increased the predicted risk of SARE, while higher PNI and albumin were protective.

The bar plot of mean absolute SHAP values (Figure 4B) quantified the overall contribution ranking of the 12 predictors. Because the final model is a linear logistic regression, each SHAP value is a linear function of the corresponding feature value (SHAPi = βi·(xi − E[xi])); accordingly, the model itself implies no non-linear or threshold effects. Individual-level force plots (Figure 4C) illustrate how the model integrates the 12 variables to produce patient-specific risk estimates: a low-risk patient (predicted probability 0.41) showed favorable dosimetric and inflammatory profiles, whereas a high-risk patient (predicted probability 0.59) was driven toward SARE by elevated rectal doses, high SII, and low PNI. The coefficients of all 12 predictors are reported in Table S5.

Overall, the SHAP analysis confirms that SARE risk is driven by a combination of moderate-dose rectal exposure (especially V30–V40), systemic inflammation (NLR, SII, PLR), and poor nutritional–immune reserve (low PNI and albumin), providing a biologically plausible and clinically informative basis for risk stratification.

## 4. Discussion

In this study, we developed and compared six machine learning models to predict clinically significant acute radiation enteritis (SARE, grade ≥2) in cervical cancer patients receiving pelvic radiotherapy. Using a comprehensive set of 48 clinical, dosimetric, and inflammatory–nutritional variables, we identified 12 key predictors and found that the logistic regression (LR) model demonstrated the most balanced performance across training, internal test, and temporal validation cohorts. Specifically, the LR model achieved AUCs of 0.854, 0.830, and 0.754, respectively, with acceptable calibration (Brier score 0.153–0.176) and positive net benefit on decision curve analysis. In contrast, more complex models such as random forest (RF) and LightGBM showed perfect discrimination in the training set (AUC = 1.000) but lower generalizability in validation, consistent with overfitting. To our knowledge, this is among the first studies to systematically integrate dosimetric, inflammatory, and nutritional parameters with multiple ML algorithms for SARE prediction and to evaluate the resulting models in a temporally independent validation cohort. Our findings confirm that SARE is a multifactorial process. The 12 selected predictors fall into three biologically and clinically relevant categories: (1) dosimetric parameters reflecting rectal and small bowel exposure (Rectal_Dmean, Rectal_V20, Rectal_V30, Rectal_V40, Bowel_Dmean, Bowel_V30); (2) systemic inflammatory indices (NLR, PLR, SII, LAR); and (3) nutritional–immune indices (albumin, PNI). This composition underscores that beyond traditional dose constraints, the host’s inflammatory and nutritional status plays a critical role in determining individual susceptibility to radiation-induced intestinal injury.

Dosimetric parameters were among the most influential predictors in our model, with Rectal_Dmean having the highest SHAP importance. This aligns with previous studies showing that rectal and small bowel dose metrics are strongly associated with acute gastrointestinal toxicity [6,7,10,11]. Notably, our analysis also identified Rectal_V20 and Bowel_V30 as significant contributors, suggesting that even intermediate-dose exposure can trigger clinically meaningful enteritis. These findings challenge the traditional focus on high-dose metrics (e.g., V50) and support the inclusion of low-to-moderate dose constraints in treatment plan optimization. The median rectal V30 and bowel V30 values observed in the non-SARE group (66% and 19%, respectively) may serve as hypothesis-generating reference points for plan evaluation; however, these values were derived retrospectively from association analyses and should not be interpreted as validated dose constraints.

Inflammatory indices (NLR, PLR, SII, LAR) were also prominent in the model. Radiation induces DNA damage and oxidative stress in intestinal epithelial cells, triggering the release of pro-inflammatory cytokines and recruitment of neutrophils and platelets [5,8]. Elevated NLR and PLR reflect a shift toward a pro-inflammatory state relative to adaptive immunity, while SII, which combines platelets, neutrophils, and lymphocytes, provides a more comprehensive measure of systemic inflammation [11,12]. In our cohort, patients who developed SARE had significantly higher NLR (median 2.59 vs. 1.94), PLR (161.9 vs. 130.5), and SII (640.3 vs. 422.5) compared to those without SARE (Table 1). LAR, a composite of lactate dehydrogenase and albumin, has been linked to tumor metabolism and nutritional reserve; its higher values in the SARE group suggest that greater inflammatory burden and poorer metabolic reserve increase toxicity risk. These findings support pre-treatment assessment of these inexpensive, routinely available blood markers to identify high-risk patients. Nutritional and immune reserve, reflected by albumin and PNI, were protective factors. Low albumin and PNI were strongly associated with SARE (both *p* < 0.001, Table 1). PNI, calculated as albumin (g/L) + 5 × lymphocyte count (10^9^/L), integrates nutritional and immune status. Malnutrition impairs mucosal repair and immune function, reducing the gut’s tolerance to radiation [8,10]. In our study, the non-SARE group had a mean PNI of 51.2, while the SARE group had 47.9, indicating that even a modest reduction in PNI may increase risk. Therefore, pre-radiotherapy nutritional assessment and intervention, such as dietary counseling, oral nutritional supplements, or even prophylactic enteral nutrition, may be reasonable for high-risk patients, although interventional studies are needed to confirm whether such measures reduce SARE incidence.

A key finding of our study is the comparison of six ML algorithms. LR outperformed complex models in terms of generalizability and calibration. While RF and LightGBM achieved perfect training AUCs (1.000), their performance dropped substantially in the internal test and temporal validation sets (RF internal test AUC 0.781, temporal validation 0.762; LightGBM internal test 0.753, temporal validation 0.757). This indicates overfitting to training data, a common pitfall when using ensemble or boosting methods with moderate sample sizes [13,14]. In contrast, LR, despite its simpler structure, maintained stable discrimination and calibration across datasets. Its Brier scores (0.128 training, 0.153 internal test, 0.176 temporal validation) were consistently low in the development data, although the calibration slope of 0.598 in temporal validation indicates that the predictions were too extreme in that cohort; DCA is therefore interpreted as showing positive net benefit over the pre-specified threshold range rather than as confirmation of clinical utility. For medical prediction models, especially those intended for clinical deployment, interpretability and robustness are as important as raw AUC [25]. LR provides transparent coefficient estimates, allowing clinicians to understand the direction and magnitude of each predictor’s effect. Therefore, we selected LR as the final model. The SHAP analysis further enhanced interpretability. The summary plot confirmed that higher rectal doses, higher inflammatory indices, and lower nutritional indices push predictions toward SARE. Because the final model is linear, SHAP values vary linearly with each feature; the SHAP dependence patterns (Figure S2) therefore reflect monotone, directionally consistent associations, which may inform personalized RT planning and pre-treatment counseling. Furthermore, the individual-level force plots demonstrate how the model integrates multiple variables to produce a patient-specific risk estimate, making it clinically actionable.

Several nomogram-based models have been published for ARE prediction [7,8,9,10,11]. For instance, Chen et al. developed a nomogram for grade ≥2 ARE with an AUC of approximately 0.78 [7]. Ma et al. reported a model for severe ARE with an AUC of 0.81 [8]. Our LR model achieved a higher test AUC of 0.830, likely due to the inclusion of both dosimetric and inflammation–nutrition parameters, as well as rigorous feature selection. More recently, Wang et al. compared three ML algorithms for ARE and reported an AUC of 0.83 for their best model [12]. Our study extends this by including temporal validation, SHAP interpretation, and a broader set of algorithms. However, direct comparison is difficult due to differences in sample size, endpoint definition (grade ≥2 vs. grade ≥3), and patient populations.

Our LR model could potentially be implemented as a web-based or spreadsheet-based calculator for pre-RT risk assessment once it has been prospectively validated. The model’s predictions are associative rather than causal: for patients with a higher predicted probability of SARE, clinicians may wish to prioritize plan review (e.g., re-evaluating rectal and bowel V30), nutritional assessment, and closer monitoring of gastrointestinal symptoms during treatment. Any intensified interventions, such as prophylactic nutritional support or modified RT techniques, should be evaluated within prospective studies before adoption into routine practice. Conversely, lower-risk patients could be managed with standard protocols, potentially avoiding unnecessary interventions.

There are also several limitations in this study. First, this was a retrospective study, which may introduce selection and information bias, such as the risk of bias with PROBAST [26]. All dosimetric predictors in this study were derived from the external-beam radiotherapy plan alone. The intracavitary brachytherapy contribution was not incorporated, because more than 80% of this retrospective cohort received conventional two-dimensional brachytherapy prescribed to reference points, for which three-dimensional rectal and bowel DVH parameters cannot be reconstructed. Second, the sample size (*N* = 363; 290 for model development) is modest for machine learning studies, particularly for complex models such as the MLP; the overfitting observed for RF and LightGBM reflects this limitation. Third, regarding validation performance and threshold choice, restricted to the sample size and at the default threshold of 0.5, sensitivity was only 0.40 (95% CI 0.18–0.62), so the model would miss many patients who develop grade ≥2 acute radiation enteritis. Fourth, dosimetry reflects external-beam radiotherapy only. The brachytherapy contribution was not incorporated because more than 80% of the cohort received two-dimensional brachytherapy prescribed to reference points, for which three-dimensional rectal and bowel DVH parameters cannot be reconstructed; Rectal_Vx, Bowel_Vx and Dmean therefore reflect external-beam rather than cumulative exposure, and the dosimetric component underestimates total rectal and bowel dose, most relevantly for rectal toxicity. Prospective multicenter studies with CT- or MRI-based brachytherapy and EQD2-based cumulative dose assessment are needed [27]. Finally, 96.9% received concurrent chemotherapy (281/290), so the findings may not extend to radiotherapy alone. Although a temporally independent cohort was used for validation, it originated from the same institution, and broader multicenter, multi-region validation is needed.

In conclusion, among the six machine learning models compared for predicting grade ≥2 acute radiation enteritis in patients with cervical cancer receiving radiotherapy, the logistic regression model showed the most favorable balance of discrimination, calibration and interpretability. The three leading contributors by mean absolute SHAP value were Rectal_Dmean, Bowel_V30 and SII. Prospective, multicenter validation, incorporating three-dimensional brachytherapy dosimetry and a prospectively defined risk threshold, is required before its clinical utility can be established.

## Figures and Tables

**Table 1:** Comparison of clinical data between SARE group and non-SARE group.

Variable	Overall, *N* = 290	Non-SARE, *N* = 204	SARE, *N* = 86	*p*-Value
Hypertension				<0.001
Yes	75 (25.86%)	38 (18.63%)	37 (43.02%)	
No	215 (74.14%)	166 (81.37%)	49 (56.98%)	
Diabetes				>0.999
No	279 (96.21%)	196 (96.08%)	83 (96.51%)	
Yes	11 (3.79%)	8 (3.92%)	3 (3.49%)	
Radiation dose				
Rectal Dmean	35.13 (2.83)	34.51 (2.63)	36.60 (2.76)	<0.001
Rectal_ V20	88.76 [81.29, 93.95]	88.96 [82.67, 93.97]	85.99 [78.29, 93.86]	0.047
Rectal_ V30	68.92 (12.72)	66.49 (11.72)	74.68 (13.20)	<0.001
Rectal_ V40	36.24 (6.49)	34.95 (6.19)	39.31 (6.17)	<0.001
Rectal_ V45	23.57 [19.36, 26.88]	22.97 [18.74, 26.23]	25.45 [21.84, 29.46]	<0.001
Rectal_ V50	8.81 [5.40, 12.97]	8.36 [5.23, 12.55]	9.55 [5.46, 15.47]	0.163
Bowel Dmean	19.87 [14.44, 23.35]	18.46 [13.41, 22.68]	22.11 [19.10, 23.80]	<0.001
Bowel_V20	49.22 [35.30, 60.90]	44.98 [31.94, 58.23]	55.79 [46.46, 63.96]	<0.001
Bowel_V30	20.72 [14.97, 28.97]	19.31 [14.27, 27.91]	26.16 [18.36, 30.24]	<0.001
Bowel_V40	7.19 [4.34, 10.80]	6.96 [4.18, 9.84]	8.22 [4.93, 12.43]	0.064
Bowel_V45	3.59 [1.94, 5.80]	3.49 [1.93, 5.30]	4.23 [1.95, 6.68]	0.254
Bowel_V50	1.17 [0.48, 2.34]	1.13 [0.51, 2.25]	1.33 [0.36, 3.06]	0.672
Radiotherapy days	42.00 [39.00, 44.00]	42.00 [39.00, 44.00]	42.00 [38.00, 45.00]	0.809
Acute radiation enteritis				
Grade 0	129 (44.48%)	129 (63.24%)	0 (0.00%)	—
Grade 1	75 (25.86%)	74 (36.27%)	0 (0.00%)	
Grade 2	69 (23.79%)	0 (0.00%)	69 (80.23%)	
Grade ≥3	17 (5.86%)	0 (0.00%)	17 (19.77%)	
WBCs	5.49 [4.48, 6.73]	5.44 [4.48, 6.34]	6.08 [4.50, 7.30]	0.093
Neutrophil	3.38 [2.59, 4.23]	3.23 [2.56, 3.97]	4.03 [2.68, 5.18]	<0.001
Lymphocyte	1.61 [1.26, 1.97]	1.66 [1.31, 2.06]	1.45 [1.16, 1.72]	<0.001
Monocyte	0.31 [0.22, 0.40]	0.31 [0.20, 0.39]	0.32 [0.23, 0.42]	0.407
HGB	117.0 [108.0, 124.0]	117.00 [108.0, 123.5]	115.0 [104.0, 126.0]	0.467
PLTs	220.0 [170.0, 270.0]	215.50 [167.5, 261.0]	227.5 [189.0, 284.0]	0.176
LDH	175.50 [154.0, 196.0]	172.50 [151.5, 190.0]	180.0 [161.0, 208.0]	0.018
Albumin	41.95 [40.20, 44.20]	42.80 [40.70, 44.55]	40.70 [38.70, 42.90]	<0.001
PLR	137.86 [104.49, 182.73]	130.48 [102.20, 168.79]	161.91 [124.10, 215.79]	<0.001
NLR	2.12 [1.54, 2.90]	1.94 [1.42, 2.69]	2.59 [1.94, 3.55]	<0.001
LMR	5.41 [4.03, 7.46]	5.78 [4.33, 8.00]	5.02 [3.48, 6.21]	0.003
SII	466.92 [308.43, 683.27]	422.53 [286.04, 591.02]	640.25 [367.36, 978.39]	<0.001
SIRI	0.61 [0.37, 0.96]	0.54 [0.32, 0.89]	0.82 [0.51, 1.26]	<0.001
PNI	50.21 (4.65)	51.19 (4.29)	47.88 (4.66)	<0.001
LAR	4.17 [3.66, 4.69]	4.00 [3.61, 4.56]	4.33 [4.08, 5.10]	<0.001
Age	57.63 (11.16)	57.31 (10.98)	58.40 (11.62)	0.461
BMI	23.19 [20.83, 25.56]	23.14 [20.90, 25.78]	23.27 [20.82, 24.97]	0.402
Tumor diameter	4.00 [3.30, 4.90]	4.00 [3.00, 4.50]	4.50 [3.90, 5.00]	<0.001
Degree of differentiation				
Moderately to poorly differentiated	148 (51.03%)	113 (55.39%)	35 (40.70%)	
Well differentiated	124 (42.76%)	77 (37.75%)	47 (54.65%)	0.026
Unknown	18 (6.21%)	14 (6.86%)	4 (4.65%)	
Histological classification				
Squamous cell carcinoma	255 (87.93%)	179 (87.75%)	76 (88.37%)	
Adenocarcinoma	28 (9.66%)	21 (10.29%)	7 (8.14%)	0.579
Others	7 (2.41%)	4 (1.96%)	3 (3.49%)	
Infiltration depth				
≥1/2	133 (45.86%)	103 (50.49%)	30 (34.88%)	0.021
<1/2	157 (54.14%)	101 (49.51%)	56 (65.12%)	
Surgery				
Yes	150 (51.72%)	115 (56.37%)	35 (40.70%)	0.021
No	140 (48.28%)	89 (43.63%)	51 (59.30%)	
Vascular tumor thrombus				
Yes	88 (30.34%)	63 (30.88%)	25 (29.07%)	0.868
No	202 (69.66%)	141 (69.12%)	61 (70.93%)	
Nerve invasion				
Yes	36 (12.41%)	24 (11.76%)	12 (13.95%)	0.748
No	254 (87.59%)	180 (88.24%)	74 (86.05%)	
Pelvic lymph node metastasis				
Yes	92 (31.72%)	61 (29.90%)	31 (36.05%)	0.528
No	197 (67.93%)	142 (69.61%)	55 (63.95%)	
Unknown	1 (0.34%)	1 (0.49%)	0 (0.00%)	
Clinical staging				
I–II	148 (51.03%)	103 (50.49%)	45 (52.33%)	0.875
III–IV	142 (48.97%)	101 (49.51%)	41 (47.67%)	
Chemotherapy				
Yes	281 (96.90%)	196 (96.08%)	85 (98.84%)	0.289
No	9 (3.10%)	8 (3.92%)	1 (1.16%)	
Chemotherapy treatment course				
<3	161 (55.52%)	108 (52.94%)	53 (61.63%)	0.219
≥3	129 (44.48%)	96 (47.06%)	33 (38.37%)	
Radiotherapy dose	50.40 [50.40, 56.00]	50.40 [50.40, 56.00]	50.40 [50.00, 56.00]	0.010
Number of radiotherapy sessions	28.00 [28.00, 28.00]	28.00 [28.00, 28.00]	28.00 [25.00, 28.00]	<0.001
Post-irradiation dose	20.00 [20.00, 28.00]	20.00 [20.00, 25.00]	25.00 [20.00, 30.00]	0.102
Number of post-loading operations	4.00 [4.00, 5.00]	4.00 [4.00, 5.00]	5.00 [4.00, 5.00]	0.441

Data are presented as median [interquartile range] or mean (standard deviation) for continuous variables, and as *n* (%) for categorical variables. Comparisons between the non-SARE group (grade 0–1 ARE, *n* = 204) and the SARE group (grade ≥2 ARE, *n* = 86) were performed using the Mann–Whitney U test, independent t-test, χ^2^ test, or Fisher’s exact test as appropriate. Significant differences (*p* < 0.05) were observed for hypertension, rectal and bowel dosimetric parameters (Dmean, V20, V30, V40, V45), inflammatory indices (NLR, PLR, SII, LAR), nutritional markers (albumin, PNI), tumor diameter, differentiation degree, infiltration depth, surgery history, radiotherapy dose, and number of radiotherapy sessions. Abbreviations: SARE, severe acute radiation enteritis; Dmean, mean dose; Vx, percentage of organ volume receiving x Gy; NLR, neutrophil-to-lymphocyte ratio; PLR, platelet-to-lymphocyte ratio; SII, systemic immune–inflammation index; LAR, lactate dehydrogenase-to-albumin ratio; PNI, prognostic nutritional index; WBCs, white blood cells; HGB, hemoglobin; PLTs, platelets; LDH, lactate dehydrogenase; BMI, body mass index.

**Table 2:** Predictive performance of six machine learning models on training, internal test, and temporal validation datasets.

Model	Dataset	Accuracy	Sensitivity	Precision	Specificity	F1 Score	AUC
SVM	Training	0.7833	0.3667	0.7857	0.958	0.5	0.7751
SVM	Test	0.7356	0.3462	0.6	0.9016	0.439	0.761
SVM	External	0.7222	0.2	0.5	0.9231	0.2857	0.599
XGBoost	Training	0.8473	0.5	0.9677	0.993	0.6593	0.9552
XGBoost	Test	0.7241	0.2692	0.5833	0.918	0.3684	0.7604
XGBoost	External	0.75	0.15	0.75	0.9808	0.25	0.6606
LightGBM	Training	1	1	1	1	1	1
LightGBM	Test	0.7356	0.5769	0.5556	0.8033	0.566	0.7528
LightGBM	External	0.7917	0.45	0.6923	0.9231	0.5455	0.7567
Logistic Regression	Training	0.7833	0.5	0.6818	0.9021	0.5769	0.8544
Logistic Regression	Test	0.7816	0.5769	0.6522	0.8689	0.6122	0.8304
Logistic Regression	External	0.7639	0.4	0.6154	0.9038	0.4848	0.7538
Random Forest	Training	1	1	1	1	1	1
Random Forest	Test	0.7126	0.3462	0.5294	0.8689	0.4186	0.7806
Random Forest	External	0.7361	0.25	0.5556	0.9231	0.3448	0.7615
MLP	Training	0.6404	0.75	0.4369	0.5944	0.5521	0.7275
MLP	Test	0.5977	0.6923	0.4	0.5574	0.507	0.7371
MLP	External	0.5972	0.6	0.3636	0.5962	0.4528	0.6163

Note: Metrics include accuracy, sensitivity, precision, specificity, F1 score and area under the receiver operating characteristic curve (AUC). All metrics were calculated at a classification threshold of 0.5 and were regenerated from a single locked prediction vector; the values in this table are definitive and are identical to those quoted in the main text. The logistic regression (LR) model achieved the highest internal test AUC (0.830) and a temporal validation AUC of 0.754, with a balanced precision–recall trade-off (F1 score = 0.612 in the internal test set). Random forest (RF) and LightGBM showed perfect training performance (AUC = 1.000) but substantial declines in validation, indicating overfitting. XGBoost, the SVM and the MLP exhibited moderate to low generalizability. Abbreviations: LR, logistic regression; MLP, multilayer perceptron; SVM, support vector machine; RF, random forest; AUC, area under the receiver operating characteristic curve.

## Data Availability

The primary data supporting the findings of this study are available from the corresponding author on reasonable request. The data supporting the findings of this study are also available in the Supplementary Materials of this article.

## Supplementary Materials

The following supporting information can be downloaded at https://www.mdpi.com/article/10.3390/jcm15218501/s1: Figure S1: Precision–recall curves of six machine learning models; Figure S2: SHAP dependence plots of the 12 predictors in the final L2-regularized logistic regression model, computed in the internal test set (*n* = 87). Each panel shows the SHAP value (contribution to the predicted log-odds of grade ≥2 acute radiation enteritis) as a function of the predictor value; β denotes the corresponding regression coefficient from Table S5. SHAP values were calculated as SHAP_i_ = β_i_·(x_i_ − E[x_i_]), where E[x_i_] is the training-set mean. Because the final model is linear, the dependence patterns are monotone and directionally consistent; Table S1: Validation cohort baseline and treatment characteristics; Table S2: Variance inflation factors; Table S3: Unrounded estimates and bootstrap intervals; Table S4: Comparative calibration performance and Brier scores of six machine learning models; Table S5: Final L2-regularized logistic regression coefficients.
