# Supplementary material for: Construction and Temporal Validation of a Machine Learning Model for Predicting Clinically Significant (Grade ≥2) Acute Radiation Enteritis After Radiotherapy for Cervical Cancer
Source: J Clin Med. 2026 Nov 1;15(21):8501. doi: 10.3390/jcm15218501 (PMC13764392; doi:10.3390/jcm15218501)
Supplement: Supplementary file 1 [file jcm-15-08501-s001.zip › Figures S1 and S2.pdf]

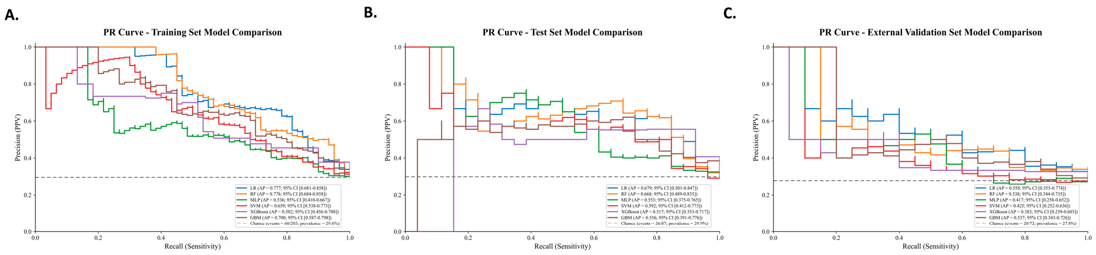

Figure S1: Precision–recall curves of six machine learning models.

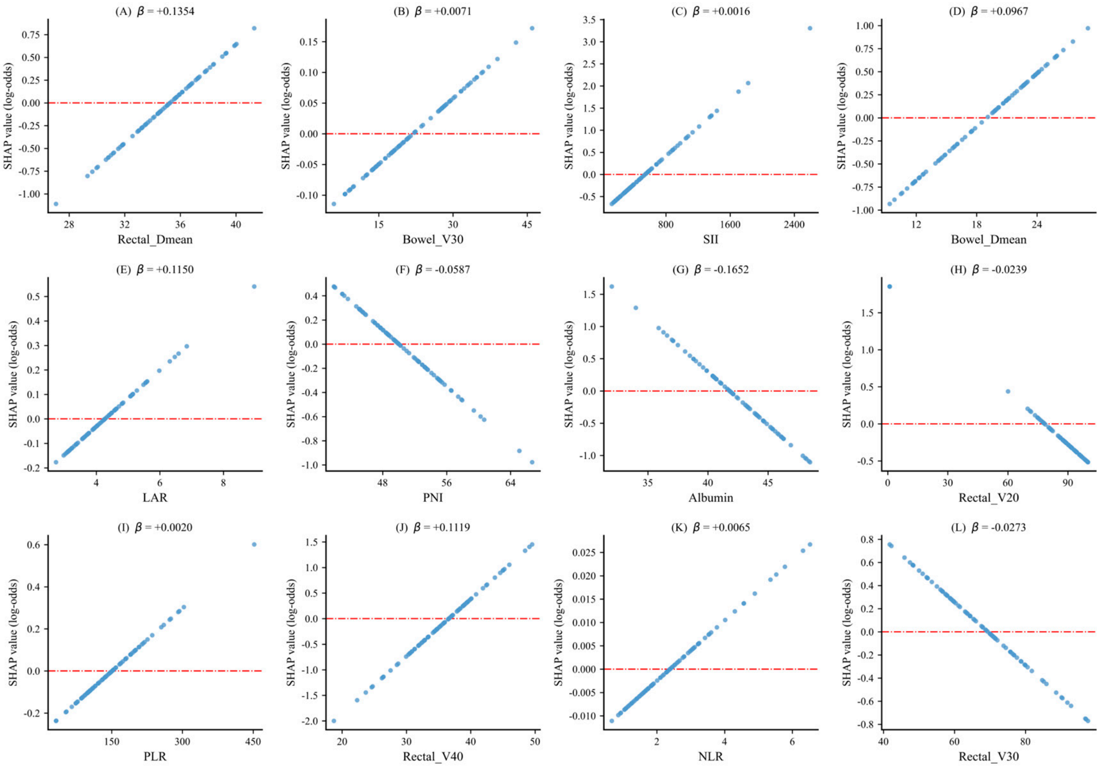

Figure S2: SHAP dependence plots of the 12 predictors in the final L2-regularized logistic regression model, computed in the internal test set (n = 87). Each panel shows the SHAP value (contribution to the predicted log-odds of grade ≥2 acute radiation enteritis) as a function of the predictor value; β denotes the corresponding regression coefficient from Table S5. SHAP values were calculated as $SHAP_i = \beta_i \cdot (x_i - E[x_i])$, where $E[x_i]$ is the training-set mean. Because the final model is linear, the dependence patterns are monotone and directionally consistent.
